# Single Gas Permeance Performance of High Silica SSZ-13 Zeolite Membranes

**DOI:** 10.3390/membranes8030043

**Published:** 2018-07-13

**Authors:** Li Liang, Meihua Zhu, Le Chen, Caijun Zhong, Yiming Yang, Ting Wu, Heli Wang, Izumi Kumakiri, Xiangshu Chen, Hidetoshi Kita

**Affiliations:** 1 Institute of Advanced Materials, State-Province Joint Engineering Laboratory of Zeolite Membrane Materials, College of Chemistry and Chemical Engineering, Jiangxi Normal University, Nanchang 330022, China; liliang@126.com (L.L.); chenle@126.com (L.C.); zhongcaijun@126.com (C.Z.); yangyimin@163.com (Y.Y.); ting.wu02@hotamail.com (T.W.); wangheliheli@163.com (H.W.); 2 Department of Environmental Science and Engineering, Graduate School Science and Engineering, Yamaguchi University, Tokiwadai 2-16-1, Ube, Yamaguchi 755-8611, Japan; izumi.k@yamaguchi-u.ac.jp (I.K.); kita@yamaguchi-u.ac.jp (H.K.)

**Keywords:** high silica SSZ-13 zeolite membrane, aluminum-free, single gas permeance, CO_2_, CH_4_

## Abstract

Continuous and high silica SSZ-13 zeolite membranes were prepared on porous mullite supports from high SiO_2_/Al_2_O_3_ ratio or aluminum-free precursor synthesis gel. Single gas permeance (CO_2_ and CH_4_) of the high silica SSZ-13 zeolite membrane was decreased with the SiO_2_/Al_2_O_3_ ratio in the precursor synthesis gel, while the ideal CO_2_/CH_4_ selectivity of the membrane was gradually increased. Moreover, effects of synthesis conditions (such as H_2_O/SiO_2_ and RNOH/SiO_2_ ratios of precursor synthesis gel, crystallization time) on the single gas permeance performance of high silica SSZ-13 zeolite membranes were studied in detail. Medium H_2_O/SiO_2_ and RNOH/SiO_2_ ratios in the initial synthesis gel were crucial to prepare the good CO_2_ perm-selective SSZ-13 zeolite membrane. When the molar composition of precursor synthesis gel, crystallization temperature and time were 1.0 SiO_2_: 0.1 Na_2_O: 0.1 TMAdaOH: 80 H_2_O, 160 °C and 48 h, CO_2_ permeance and ideal CO_2_/CH_4_ selectivity of the SSZ-13 zeolite membrane were 0.98 × 10^−7^ mol/(m^2^·s·Pa) and 47 at 25 °C and 0.4 MPa. In addition, the SiO_2_/Al_2_O_3_ ratio of the corresponding SSZ-13 zeolite was 410 by X-ray fluorescence spectroscopy.

## 1. Introduction

As a high-quality, clean fuel and important chemical raw materials, natural gas has attracted a lot of attention recently. Global consumption of natural gas was projected to increase to 182 trillion m^3^ in 2030 [1]. CO_2_ content of natural gas was high in general, which could have a great influence on the combustion heat of natural gas. In addition, the high CO_2_ content natural gas would corrode the steel pipeline and increase the transportation equipment costs. Generally, specifications for natural gas require a CO_2_ concentration below 2–3% [2]. 

Currently, amine adsorption and cryogenic distillation are the main technologies for CO_2_ capture and separation, which are the energy-intensive and cost-intensive processes [3]. Membrane separation was expected to be a novel and energy efficient technology for CO_2_ capture and separation, which was no need for sorbent regeneration or desorption [4]. Baker et al. reported that polymeric membranes could separate CO_2_ from natural gas [5]. However, the high CO_2_ pressure could plasticize polymeric membranes and decrease separation performance of polymeric membranes [6]. Because the membrane had the inherent trade-off property for gas separation, it was difficult to obtain the high permeation and selectivity polymeric membrane [7]. Inorganic membranes have good hydrothermal stability, mechanical strength, and chemical resistance, which are promising candidates for gas separation [8]. Recently, zeolite membranes, such as zeolite T, DDR, MFI, CHA, and metal-organic framework (MOF) membranes have gained much attention for natural gas purification [8,9,10,11,12,13,14,15,16,17,18,19,20,21,22,23]. 

SSZ-13 zeolite had a unique three-dimensional structure with eight-membered rings and intersecting channels with a ring diameter of 0.37 nm × 0.42 nm [24]. SSZ-13 zeolite membranes had good selectivity and permeance for CO_2_/CH_4_ separation [3,21,22]. In particular, the high-silica zeolite membrane had low polarity and few non-zeolitic molecular trafficking pathways [25,26,27]. In addition, the high-silica SSZ-13 membrane was successfully prepared on α-alumina hollow fibers, the optimal CO_2_ permeance and CO_2_/CH_4_ separation of the membrane were 2.5 × 10^−7^ mol/(m^2^·s·Pa) [28]. In order to prepare a high silica SSZ-13 zeolite membrane, the SiO_2_/Al_2_O_3_ ratio of the initial synthesis gel was up to 40 in our previous study [3]. Kida et al. had successfully prepared the pure silica CHA-type zeolite membrane, and the synthesis procedure was complicated; for example, the H_2_O/SiO_2_ ratio of initial synthesis gel was only 5.7 [21]. A low H_2_O/SiO_2_ ratio means high costs of membrane preparation, which would affect the industrial application of an SSZ-13 zeolite membrane.

High silica SSZ-13 zeolite membranes were prepared from the aluminum-free or high SiO_2_/Al_2_O_3_ ratio precursor synthesis gel by secondary hydrothermal synthesis, and the H_2_O/SiO_2_ ratio of the precursor synthesis gel was from 20 to 80 in this work. In order to optimize synthesis conditions of high silica SSZ-13 zeolite membranes, influences of synthesis conditions (the RNOH/SiO_2_ and H_2_O/SiO_2_ ratios of the initial synthesis gel, crystallization time) on growth and single gas separation performance of high silica SSZ-13 zeolite membranes were investigated in details.

## 2. Materials and Methods

### 2.1. Preparation Procedure of SSZ-13 Zeolite and High Silica SSZ-13 Membranes

A high silica SSZ-13 zeolite membrane was synthesized on the porous mullite tubes (length: 10 cm, out diameter: 12 mm, inner diameter: 9 mm, thickness: 1.5 mm, average pore size: 1.3 μm, porosity: 30–40%, Nikkato Corp., Tokyo, Japan) by secondary hydrothermal synthesis. The synthesis procedure of SSZ-13 zeolite seed was as following, NaOH (96%, Junzheng, Wuhai, China), N, N, *N*-trimethyl-1-adamantammonium hydroxide (TMAdaOH, 25%, Ankai, Tokyo, Japan), Al(OH)_3_ (99%, Wako, Tokyo, Japan) were mixed by deionized water and stirred the mixture, and then colloidal silica (Ludox TM-40, Sigma-Aldrich, St. Louis, MO, USA) was slowly added into the mixture while continuously stirring. The molar composition of SSZ-13 zeolite seed synthesis gel was SiO_2_: 0.1 Na_2_O: 0.025 Al_2_O_3_: 0.1 TMAdaOH: 44 H_2_O. The mixture was aged at room temperature for 6 h, and transferred to a Teflon-lined autoclave and placed into an oven at 160 °C for 96 h. The SSZ-13 zeolite seeds were centrifuged with deionized water after hydrothermal synthesis and calcined at 550 °C for removing an organic template. The mullite tube was seeded by the home-made SSZ-13 zeolite seeds by rub-coating, and the molar composition of initial synthesis gel for SSZ-13 zeolite membrane was SiO_2_: 0.1 Na_2_O: *x* Al_2_O_3_: *y* TMAdaOH: *z*H_2_O (*x* = 0–0.025, *y* = 0.05–0.15, *z* = 20–80). The preparation procedure of the high silica SSZ-13 membrane was similar to our previous study [3]. NaOH, TMAdaOH, and Al(OH)_3_ were added into deionized water, and stirred to form a clear solution. Colloidal silica was slowly added to the clear solution, and the mixtures were aged 6 h at room temperature while continuously stirring. Thereafter, the precursor synthesis gel and seeded tubes were transferred to a Teflon-lined autoclave and placed into an oven at 160 °C for 24–78 h. The membrane was washed with deionized water, and TMdaOH of the membrane was removed at 550 °C for 10 h. Moreover, the synthesis procedure of different SiO_2_/Al_2_O_3_ ratio SSZ-13 zeolite was identical with the SSZ-13 zeolite membrane except a seeded mullite tube, and the SSZ-13 zeolite seed content in the precursor synthesis gel was 0.5 wt %.

### 2.2. Characterization and Single-Gas Permeation

Surface morphology and thickness of membranes were observed by field emission scanning electron microscopy (FE-SEM, SU8020, Hitachi, Tokyo, Japan) at acceleration voltages of 3–10 kV. X-ray diffraction (XRD, Ultima IV, Rigaku, Tokyo, Japan) was used to identify the crystal phases of the SSZ-13 zeolite and membrane, and the test condition was Cu-Kα radiation with 2θ from 5° to 45º. SiO_2_/Al_2_O_3_ ratio of SSZ-13 zeolite was characterized by X-ray fluorescence spectroscopy (XRF, S4 PIONEER, Bruker, Billerica, MA, USA).

**Figure 1:**
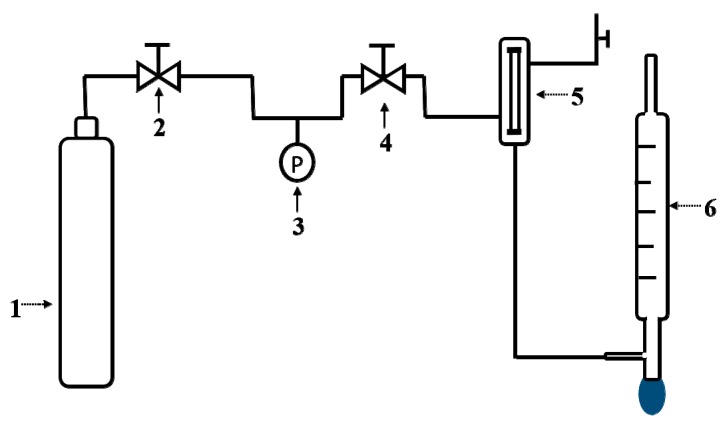
Single gas permeation measurements: (1) cylinder; (2) reducing valve; (3) pressure gauge; (4) counterbalance valve; (5) membrane module; (6) soap bubble flowmeter.

Permeance and ideal gas selectivity (CO_2_ and CH_4_) of high silica SSZ-13 zeolite membranes were tested by single gas permeation measurements at room temperature and 0.4 MPa. As shown in Figure 1, SSZ-13 zeolite membranes were mounted in a stainless steel module, and each end of the membrane was installed with silicone O-rings and two stainless steel rings. The single gas permeance (*P_i_*) was calculated by Equation (1):

*P_i_* = *n_i_*/(*A*∙Δ*P*) (1)

where *n_i_* (mol/s) was the membrane flux of component *i*, Δ*P* (Pa) was the pressure drop, and *A* (m^2^) was the effective surface area of the SSZ-13 zeolite membrane. The relevant ideal selectivity (S) of membranes was calculated with the ratio of CO_2_ and CH_4_ permeance as Equation (2):

(2) $$S=P_{CO_{2}}/P_{CH_{4}}$$

## 3. Results and Discussion

### 3.1. Effect of the SiO_2_/Al_2_O_3_ Ratio

The water adsorption property of zeolite membrane greatly depended on the SiO_2_/Al_2_O_3_ ratio of membrane layer, and the hydrophobicity of the zeolite membrane was increased with the SiO_2_/Al_2_O_3_ ratio. Generally, high silica zeolite membrane had few defects, pinholes and a good gas separation performance [20,29], and Si-CHA zeolite membrane had better vapor resistance than the topologically analogous SSZ-13 zeolite membrane [21]. In order to investigate effects of SiO_2_/Al_2_O_3_ ratio on the growth and gas separation performance of SSZ-13 zeolite membrane, which were prepared from different SiO_2_/Al_2_O_3_ ratio precursor synthesis gels (SiO_2_/Al_2_O_3_ = 40~∞). The crystallization time and temperature of the membrane were 48 h and 160 ˚C. Molar composition of the synthesis gel was SiO_2_: 0.1 Na_2_O: *x*Al_2_O_3_: 0.1 TMAdaOH: 80 H_2_O (*x* = 0, 0.005, and 0.025). CO_2_ and CH_4_ permeation results of these membranes were given in Table 1. It is noted that the preparation procedure of the corresponding high silica SSZ-13 zeolite was identical with the high silica SSZ-13 zeolite membrane, and the SiO_2_/Al_2_O_3_ ratio of corresponding high silica SSZ-13 zeolite was characterized by XRF. XRF results suggested that the SiO_2_/Al_2_O_3_ ratio of the SSZ-13 zeolite membrane was increased with the SiO_2_/Al_2_O_3_ ratio of the precursor synthesis gel, and SiO_2_/Al_2_O_3_ ratios of these membranes M-1, M-2 and M-3 were 31, 115 and 410, respectively. Because the SiO_2_/Al_2_O_3_ ratio of seed crystals was 40, even though the membrane M-3 was prepared from the aluminum-free precursor gel, the SiO_2_/Al_2_O_3_ ratio of the membrane was 410 for the aluminum-containing SSZ-13 zeolite crystals. 

**Figure 2:**
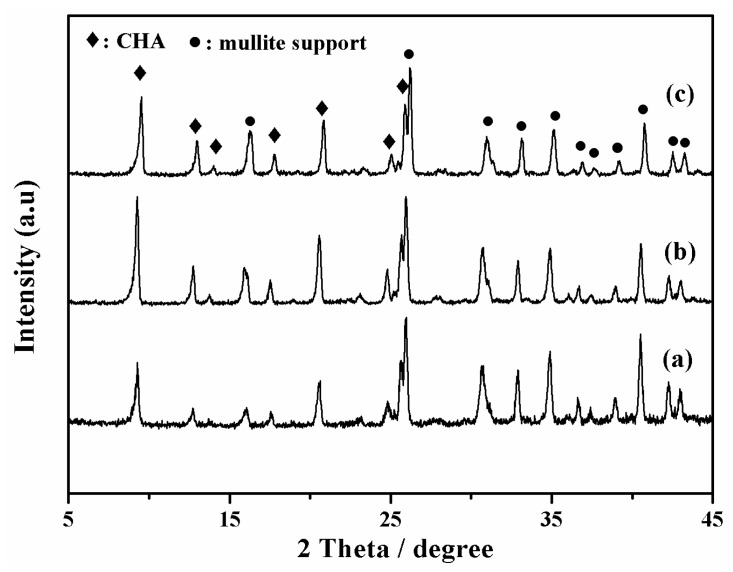
XRD patterns of high silica SSZ-13 zeolite membranes with different SiO_2_/Al_2_O_3_ ratio; (**a**) M-1, SiO_2_/Al_2_O_3_ = 40; (**b**) M-2, SiO_2_/Al_2_O_3_ =200; (**c**) M-3, SiO_2_/Al_2_O_3_ = ∞.

**Figure 3:**
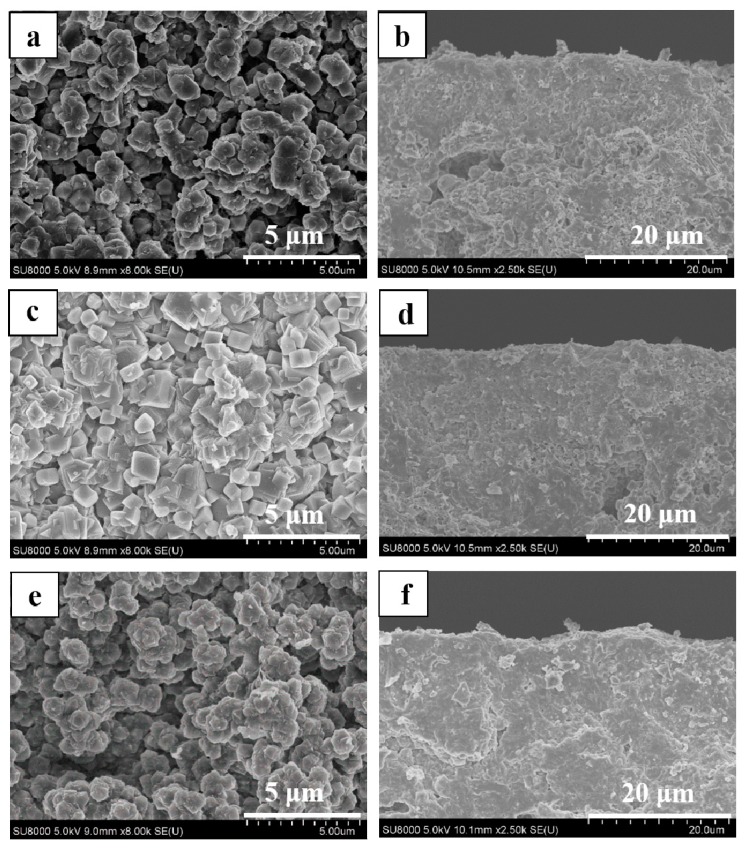
Surface and cross-sectional SEM images of high silica SSZ-13 zeolite membranes with different SiO_2_/Al_2_O_3_ ratio (**a**,**b**) M-1, SiO_2_/Al_2_O_3_ = 40; (**c**,**d**) M-2, SiO_2_/Al_2_O_3_ =200; (**e**,**f**) M-3, SiO_2_/Al_2_O_3_ = ∞.

As shown in Figure 2, all membranes (M-1, 2 and 3) had typical CHA zeolite and mullite diffraction peaks by XRD patterns, which suggested that high silica SSZ-13 zeolite membranes were successfully prepared from high SiO_2_/Al_2_O_3_ ratio or aluminum-free precursor synthesis gel in this work. The intensity of typical CHA zeolite diffraction peaks of membranes M-2 and M-3 were higher than that of membrane M-1. In addition, Figure 3 showed surface and cross sectional SEM images of membrane M-1, M-2 and M-3. Crystal layer thicknesses of SSZ-13 zeolite membranes were independent of the SiO_2_/Al_2_O_3_ ratio in the precursor synthesis gel compact crystal layers, which was consistent with the previous reference [20]. Compactness of membrane M-2 and M-3 was better than the membrane M-1 (Figure 3a,c,e).

Gas separation performance of zeolite membranes was sensitive to non-zeolite defects in the zeolite membrane selective layer [30]. As shown in Table 1, CO_2_ permeance of SSZ-13 membranes were decreased with the SiO_2_/Al_2_O_3_ ratio in the precursor synthesis gel, while the CO_2_/CH_4_ selectivity of as-synthesized SSZ-13 zeolite membranes was gradually increased, which could be attributed to the fact that the membrane had few defects with the SiO_2_/Al_2_O_3_ ratio [20]. The high SiO_2_/Al_2_O_3_ ratio precursor gel was a favor for preparing defect-free zeolite membrane, and the membrane M-3 had a better ideal selectivity than membranes M-1 and 2, which had fully justified XRD patterns and SEM images (Figure 2 and Figure 3). CO_2_ permeance and CO_2_/CH_4_ ideal selectivity of the membrane M-3 were 0.98 × 10^−7^ mol/(m^2^·s·pa) and 47, respectively. Therefore, the high silica SSZ-13 zeolite membrane was successfully prepared from aluminum-free precursor synthesis gel and showed ideal CO_2_/CH_4_ selectivity in this work. 

### 3.2. Effect of H_2_O/SiO_2_ Ratio 

Generally, the Si-CHA or high silica SSZ-13 zeolite membrane were prepared from the concentrated synthesis gel, for example, H_2_O/SiO_2_ ratio of initial synthesis gel was only 5.7 [21]. In addition, H_2_O/SiO_2_ ratio had great effects on the concentration of each component in precursor synthesis gel, including the alkalinity of synthetic mixture. A medium alkaline environment was conducive to the growth of a defect-free zeolite membrane, but the zeolite crystals would be dissolved by strong alkalinity [31]. As presented in Table 1, high silica SSZ-13 zeolite membranes were prepared from a different H_2_O/SiO_2_ ratio and aluminum-free precursor synthesis gels (M-3, 4, 5, and 6), whose molar ratio was 1.0 SiO_2_: 0.1 Na_2_O: 0.1 TMAdaOH: *z*H_2_O in this study (*z* = 20–80). 

**Figure 4:**
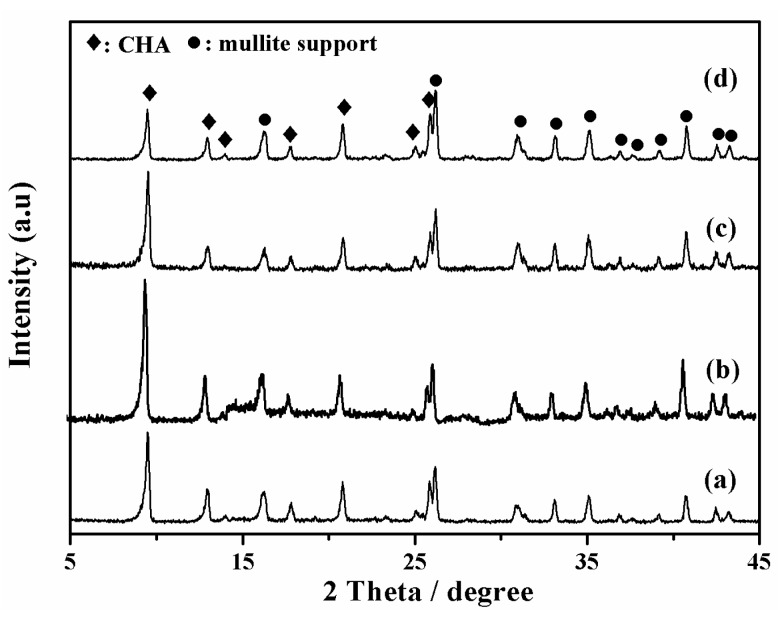
XRD patterns of high silica SSZ-13 zeolite membranes with different H_2_O/SiO_2_ ratio, (**a**) M-4, H_2_O/SiO_2_ = 20; (**b**) M-5, H_2_O/SiO_2_ = 40; (**c**) M-6, H_2_O/SiO_2_ = 60; (**d**,**b**) M-3, H_2_O/SiO_2_ = 80.

**Figure 5:**
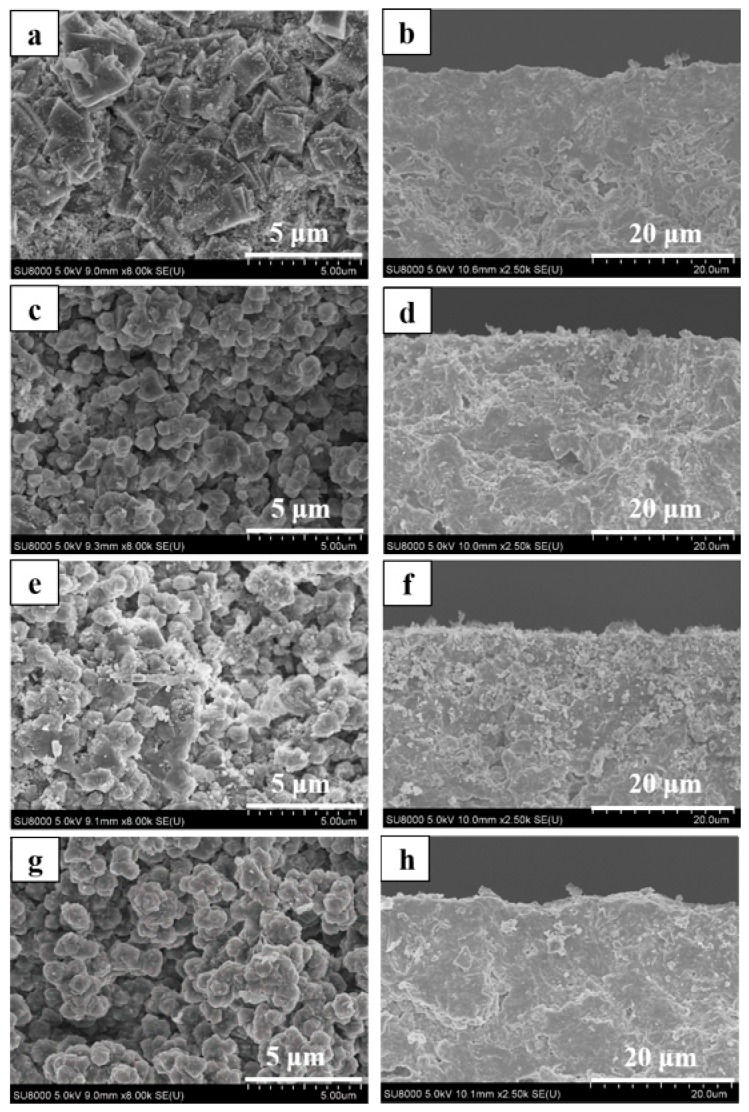
Surface and cross-sectional SEM images of high silica SSZ-13 zeolite membranes with different H_2_O/SiO_2_ ratio, (**a**,**b**) M-4, H_2_O/SiO_2_ = 20; (**c**,**d**) 40, H_2_O/SiO_2_ = 40; (**e**,**f**) M-6, H_2_O/SiO_2_ = 60; (**g**,**h**) M-3, H_2_O/SiO_2_ = 80.

XRD patterns of SSZ-13 zeolite membranes (M-3, 4, 5, and 6) were shown in Figure 4. There were no specific orientation and other zeolite or amorphous impurities of these membranes, indicating that these membranes had typical CHA topology. Figure 5 presented surface and cross-sectional SEM images of these membranes, when the H_2_O/SiO_2_ ratio in precursor synthesis gel was gradually increased, and the crystal morphology was changed from cubic crystals to spherical crystals (Figure 5a,c,e). Because there were too many nutrients for nuclei formation and zeolite growth with concentrated synthesis gel (H_2_O/SiO_2_ = 20), there were plenty of fine SSZ-13 zeolite covered on the membrane surface (Figure 5a). In addition, the thickness of membrane layer was independent of the H_2_O/SiO_2_ ratio in precursor synthesis gel. From the viewpoint of the balance of permeance and selectivity, when the H_2_O/SiO_2_ ratio in the precursor synthesis gel was 80, the membrane M-3 had a good single gas permeance in this work. 

### 3.3. Effect of TMAdaOH/SiO_2_ Ratio 

Table 1 summarized the single gas permeance of high silica SSZ-13 zeolite membranes (M-4, 7, and 8), which were prepared from different TMAdaOH/SiO_2_ ratio initial synthesis gel (1.0 SiO_2_: 0.1 Na_2_O: *y* TMAdaOH: 20 H_2_O, *y* = 0.05, 0.10, and 0.15). Both permeance and selectivity of these high silica SSZ-13 zeolite membranes were improved with the TMAdaOH/SiO_2_ ratio in precursor synthesis gel, and the permeance and selectivity of the membrane M-8 were slightly decreased at extremely high TMAdaOH/SiO_2_ ratio in precursor gel (RNOH/SiO_2_ = 0.15).

**Figure 6:**
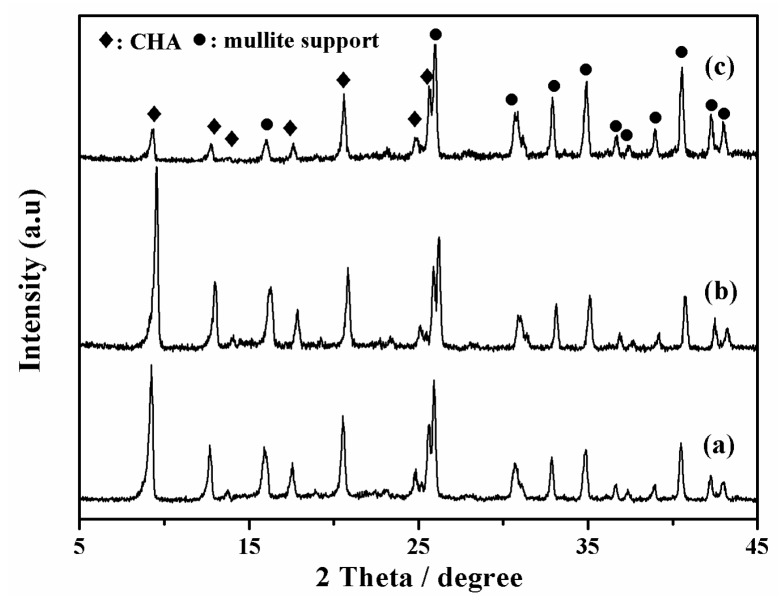
XRD patterns of high silica SSZ-13 zeolite membranes with different TMAdaOH/SiO_2_ ratio, (**a**) M-7, TMAdaOH/SiO_2_ = 0.05; (**b**) M-4, TMAdaOH/SiO_2_ = 0.10; (**c**) M-8, TMAdaOH/SiO_2_ = 0.15.

**Figure 7:**
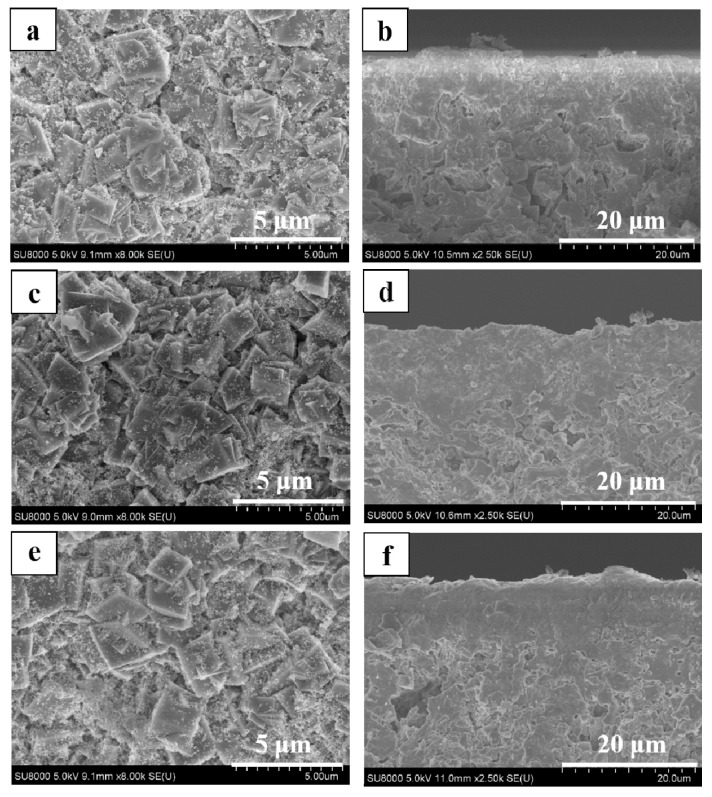
Surface and cross-sectional SEM images of high silica SSZ-13 zeolite membranes with different TMAdaOH/SiO_2_ ratio, (**a**,**b**) M-7, TMAdaOH/SiO_2_ = 0.05; (**c**,**d**) M-4, TMAdaOH/SiO_2_ = 0.10; (**g**,**h**) M-8, TMAdaOH/SiO_2_ = 0.15.

In addition, XRD patterns and SEM images of high silica SSZ-13 zeolite membrane with different TMAdaOH/SiO_2_ were shown in Figure 6 and Figure 7. Clearly, these membranes had typical CHA diffraction peaks, when the TMAdaOH/SiO_2_ ratio in precursor gel was 0.15, typical CHA zeolite diffraction peaks of the membrane M-8 were weak. The crystal size was increased with increasing RNOH/SiO_2_ ratio, and the fine SSZ-13 zeolite crystals on the membrane surface were gradually increased with increasing TMAdaOH/SiO_2_ ratio. Moreover, the membrane M-4 had a better single gas permeance performance than membranes M-7 and M-8, which were consistent with XRD patterns and SEM images. Hence, a medium-level TMAdaOH/SiO_2_ ratio of the precursor synthesis gel was critical of the preparation of high silica SSZ-13 zeolite membrane from aluminum-free synthesis gel.

### 3.4. Effect of Synthesis Time

Effects of synthesis time on SSZ-13 zeolite membranes performance were investigated in this work, and the single gas permeance of high silica SSZ-13 zeolite membranes (M-9, 10, 4 and 11) was shown in Table 1. 

**Figure 8:**
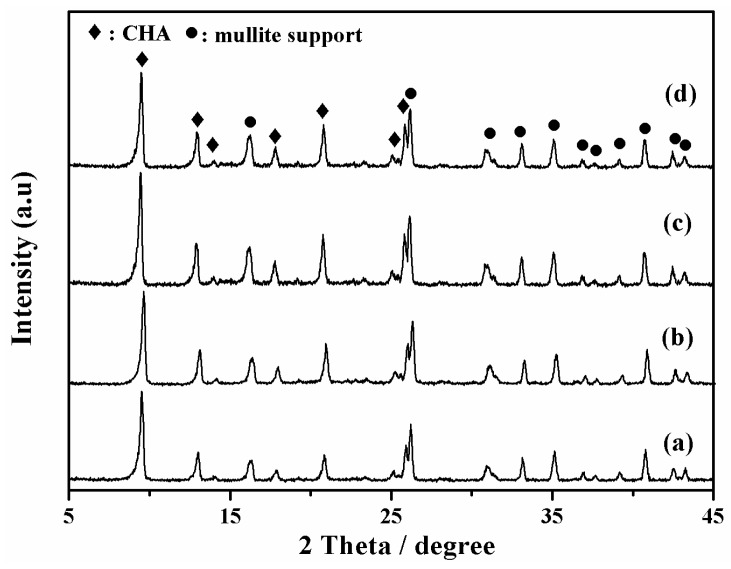
XRD patterns of high silica SSZ-13 zeolite membrane with different synthesis time, (**a**) M-9, 24 h; (**b**) M-10, 36 h; (**c**) M-4, 48 h; (**d**) M-11, 72 h.

**Figure 9:**
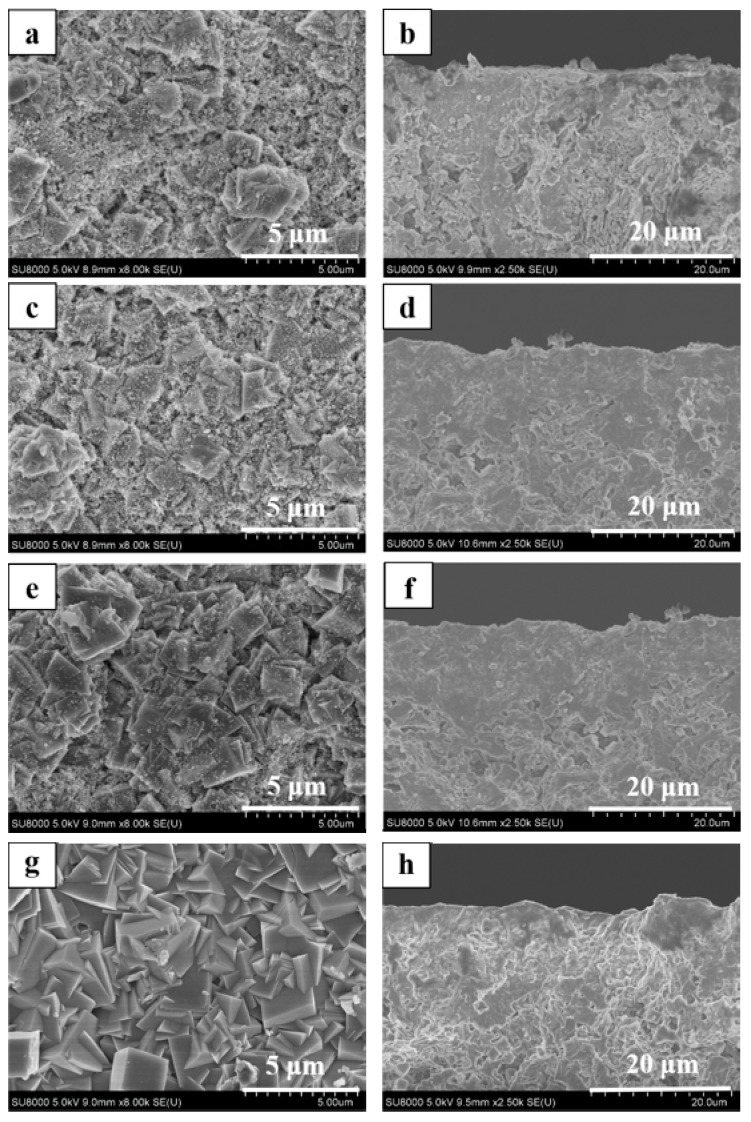
Surface and cross-sectional SEM images of high silica SSZ-13 zeolite membrane with different synthesis time, (**a**,**b**) M-9, 24 h; (**c**,**d**) M-10, 36 h; (**e**,**f**) M-4, 48 h; (**g**,**h**) M-11, 72 h.

Figure 8 showed the XRD patterns of high silica SSZ-13 zeolite membranes. All membranes had typical CHA zeolite diffraction peaks. SEM images of SSZ-13 zeolite membranes prepared with different synthesis times were shown in Figure 9. There were some fine particles on the membrane surface at 24 h, 36 h and 48 h, and the number of particles on the membrane surface was gradually decreased (Figure 9a,c,e). The crystal layer became dense, and the fine particles disappeared as the synthesis time increased to 72 h (Figure 9g,h). When the synthesis time was 48 h, the membrane M-4 showed the best single gas permeance and ideal selectivity in this work. 

In addition, comparison of ideal selectivity (S_CO2/CH4_) through SSZ-13 zeolite membrane in literature was summarized in Table 2. As shown in Table 2, good single gas permeance performance and high silica SSZ-13 zeolite membrane were successfully prepared from the aluminum-free and high H_2_O/SiO_2_ ratio precursor synthesis gel. 

## 4. Conclusions

A high silica SSZ-13 zeolite membrane had been successfully fabricated from an aluminum-free precursor synthesis gel using a secondary growth method. The SiO_2_/Al_2_O_3_ ratio of the high silica SSZ-13 zeolite membrane was up to 410 by XRF characterization. The ideal CO_2_/CH_4_ selectivity of as-synthesized SSZ-13 zeolite membranes was gradually increased with SiO_2_/Al_2_O_3_ ratio. The optimal molar composition of precursor synthesis gel, crystallization temperature and time were 1.0 SiO_2_: 0.1 Na_2_O: 0.1 TMAdaOH: 80 H_2_O, 160 °C and 48 h in this work. CO_2_ permeances and CO_2_/CH_4_ selectivity were 0.98 × 10^−7^ mol/(m^2^ s pa) and 47 at 25 °C and 0.4 MPa. 

## Figures and Tables

**Table 1:** Effects of molar composition of precursor synthesis gel and synthesis time on single gas permeance and ideal selectivity of SSZ-13 zeolite membranes (25 °C, 0.4 MPa).

No.	Molar Composition of Precursor Synthesis Gel			Synthesis Time (h)	Gas Permeance		S_CO2/CH4_
	SiO_2_/Al_2_O_3_	H_2_O/SiO_2_	TMAdaOH/SiO_2_		CO_2_ × 10^7^ (mol/(m^2^∙s∙Pa))	CH_4_ × 10^9^ (mol/(m^2^∙s∙Pa))	
M-1	40	80	0.10	48	6.77	55.10	12
M-2	200	80	0.10	48	1.41	4.67	30
M-3	∞	80	0.10	48	0.98	2.10	47
M-4	∞	20	0.10	48	9.20	46.00	20
M-5	∞	40	0.10	48	0.73	5.55	13
M-6	∞	60	0.10	48	1.00	5.20	19
M-7	∞	20	0.05	48	4.15	32.00	13
M-8	∞	20	0.15	48	8.20	50.00	16
M-9	∞	20	0.10	24	8,50	121.00	7
M-10	∞	20	0.10	36	4.94	38.00	13
M-11	∞	20	0.10	72	4.05	44.00	9

Note: Molar composition of precursor synthesis gel: as SiO_2_: 0.1 Na_2_O: *x* Al_2_O_3_: *y* TMAdaOH: *z*H_2_O (*x* = 0–0.025, *y* = 0.05–0.15, *z* = 20–80). Synthesis temperature: 160 °C.

**Table 2:** Comparison of preparation conditions and single gas permeance performance of SSZ-13 zeolite membranes in literature.

Molar Ratio of Precursor Synthesis Gel		Pressure Drop (MPa)	Single Gas Permeance Performance		Reference (-)
H_2_O/SiO_2_	SiO_2_/Al_2_O_3_		CO_2_ Permeance × 10^7^ (mol/(m^2^∙s∙Pa))	S_CO2/CH4_	
80	∞	0.40	0.98	47	This work
44	40	0.05	1.00	11	[19]
42	50	0.60	3.00	20	[32]
80	40	0.20	2.00	360	[3]
5.7	∞	0.30	15.00	45	[21]
